# Self Standing Mats of Blended Polyaniline Produced by Electrospinning

**DOI:** 10.3390/nano11051269

**Published:** 2021-05-12

**Authors:** Antonio Fotia, Angela Malara, Emilia Paone, Lucio Bonaccorsi, Patrizia Frontera, Giulia Serrano, Andrea Caneschi

**Affiliations:** 1 Department of Information Engineering, Infrastructures and Sustainable Energy, Mediterranea University of Reggio Calabria, Via Graziella Loc Feo di Vito, 89134 Reggio Calabria, Italy; antonio.fotia@unirc.it; 2 Department of Civil, Energy, Environment and Material Engineering, Mediterranea University of Reggio Calabria, Via Graziella Loc Feo di Vito, 89134 Reggio Calabria, Italy; emilia.paone@unirc.it (E.P.); lucio.bonaccorsi@unirc.it (L.B.); patrizia.frontera@unirc.it (P.F.); 3 Consorzio Interuniversitario per la Scienza e la Tecnologia dei Materiali (INSTM), 50121 Firenze, Italy; giulia.serrano@unifi.it (G.S.); andrea.caneschi@unifi.it (A.C.); 4 Department of Industrial Engineering—DIEF, University of Florence, Via di S. Marta 3, 50139 Firenze, Italy

**Keywords:** polyaniline, electrospinning, graphene oxide, iron oxide

## Abstract

Conducting nanofibers of polyaniline (PANI) doped with camphor-10-sulfonic acid (HCSA) and blended with different polymers, such as polymethyl methacrylate (PMMA) and polyvinyl acetate (PVAc), have been fabricated using the electrospinning technique. Scanning electron microscopy (SEM) and thermal gravimetric analysis (TGA) were utilized to characterize the morphology and the thermal stability of PANI-blended fibers. An extensive study was performed to understand the copolymer influence on both the structural and surface properties of the realized conductive thin films. Samples main electrical characteristics, as conductivity, specific capacitance and electrochemical performances were tested. The better mats were obtained with the use of PVAc copolymer, which showed a conductivity value two orders of magnitude higher than the PMMA system. Aiming at further improving the electrochemical features of these blended mats, hybrid fibers based on PANI/PVAc/graphene oxide and PANI/PVAc/iron oxide were also produced and characterized. The obtained mats were potentially addressed to numerous practical fields, including sensors, health applications, smart devices and multifunctional textile materials.

## 1. Introduction

Electrospinning is a process able to produce long fibers with a nanometric diameter [1]. During the process, due to a high applied voltage, a polymeric jet is elongated and deposited as continuous fibers over a collector. Large surface area, mechanical strength, surface functionality, flexibility and shape adaptability are some of the characteristics that make fibrous textiles interesting materials for new and traditional applications [2]. Indeed, a variety of electrospun nanofibers can be used in numerous fields, such as catalysis [3], sustainable exploitation of energy [4,5,6], energy storage [7,8], biotechnology [9], environmental engineering [10] and sensory and electronic devices [11,12,13,14,15]. To these purposes, fibers of organic, inorganic and hybrid composite materials can be easily produced and their properties opportunely tuned. In particular, the polymeric precursor, which can be of conductive or non-conductive nature, can play a pivotal role in the definition of fiber mat characteristics. When conductive polymers are electrospun, resulting fibers are endowed with superior electrical and optical properties, even comparable with those of metals and inorganic semiconductors [16], thus allowing their use in specific fields, where those properties are strictly required. Moreover, it should be also highlighted that mats or thin films in a nanofiber form have some advantages compared to bulk samples, since electrical and optical properties of thin films composites are closely related to the morphology of blended polymers, and easily tuned by the optimization of both film thickness and polymer concentration [16].

Conductive nanostructured polymers possess highly attractive characteristics, such as an easily controllable bandgap, high mechanical flexibility, anticorrosive properties and greater biocompatibility than that of many other inorganic materials. Additionally, these polymers in their neutral state have conductivity typically ranging from 10^−10^ to 10^−5^ S/cm and it can be even increased to 10^−2^–10^−5^ S/cm upon doping [17,18,19].

Among the various conductive polymers, polyaniline (PANI) is one of the most investigated because its electrical properties can be controlled by oxidation and protonation states. PANI can be found in three different forms: completely reduced (leucoemeraldine), half-oxidized (emeraldine) and completely oxidized (pernigraniline) [20,21]. The manufacturing of neat PANI nanofibers by electrospinning has been a great challenge for researchers. Production difficulties are mainly due to the poor solubility in common solvents, thus requiring proper doping with organic acids, like camphor-10-sulfonic acid (HCSA), aiming to increase its solubility and therefore the electrospinnability of the solution [21]. Furthermore, PANI is available only in the low molecular weight form and this implies an insufficient elasticity of the solution to be spun in a fiber form [22,23]. High molecular weight insulating polymers are used to make the solution more elastic. In the mixture of PANI with PEO or PS, the conductivity of blended fibers increased exponentially with the increase of the weight percent of doped polyaniline.

Yuxi et al. [24] fabricated electrospun fibers of PANI doped with HCSA blended with polyethylene oxide (PEO) or PMMA, observing that the conductivity of the PANI-blended fibers increased exponentially with the weight percent of doped polyaniline. In particular, 67% and 25% of PANI were blended with PEO and PMMA, respectively. The increase of PANI content improved the conductivity of mats but had a detrimental effect in obtaining continuous and coherent mats. In fact, the formation of beads mixed to fibers was often reported and, although numerous methods have been proposed to suppress beads formation, such as the addition of salts [25] or stabilizing agents [26], this composite morphology exhibited enhancements especially in sensor applications. PANI blended with poly(vinyl butyral) (PVB) and poly(ethylene oxide) (PEO) nanofibers, with a suitable proportion of beads, showed an improved adhesion to the electrode, a better electrical contact and improved sensing properties compared with beads’ free ones [27]. Therefore, the real challenge is the optimization of PANI-blended polymer solutions for electrospinning, to produce enhanced fibers suitable for many different applications. Recently, polyvinylpyrrolidone (PVP)/PEO/PANI multicomponent nanofibers were obtained using a special spinneret demonstrating a great potential for fibers production mainly addressed to smart textiles and sensors [28]. Obviously, the presence of non-conductive polymers changed the physical–chemical characteristics of the synthesized fibers.

As known, several parameters including process parameters (flow rate, applied voltage and distance needle tip to collector) solution properties (type and concentration of solvent, viscosity and dielectric constant) and environmental conditions (humidity and temperature) affect the fibrous morphology [11]. In this study, the effects of solution properties on the production of PANI-blended nanofibers were investigated. Two auxiliary polymers (PMMA and PVAc) were used to electrospin polyaniline. By varying their molecular weight and their concentration in the raw solution, blended systems with high polyaniline content were obtained. The choice of these copolymers was related to their macroscopic features, namely good mechanical properties, easy solubility and low cost, which made them eligible for simple fabrication of devices such as a supercapacitor, sensor, biomedical and corrosion applications [29,30]. Regarding other investigations, the novelty of this work is focused on the use of high content of PANI in blended systems, specifically designed for the electrospinning process and able to preserve PANI high electrical properties. Indeed, the possibility to obtain mats of conductive polymers will allow it to extend and improve their use in numerous innovative fields, such as health applications, smart devices and multifunctional textile materials [31].

It was found that among the two investigated polymers, the one that least depressed the conductivity characteristics of polyaniline was PVAc; furthermore, owing to improve the electrochemical features, the effect of graphene and iron oxides addition in the PANI/PVAc blended system was explored.

## 2. Materials and Methods

Polyaniline (emeraldine base) (average Mw = 100,000), camphor-10-sulfonic acid (HCSA), poly(methyl methacrylate) (average Mw = 996,000), poly(vinyl acetate) (average Mw = 100,000 and Mw = 500,000), chloroform, ammonium acetate, graphene oxide and iron oxide were purchased from Sigma–Aldrich ( St. Louis, MO, USA).

### 2.1. Nanofibers Fabrication

Doping of PANI (EB—emeraldine base) into PANI salt (ES—emeraldine salt) was performed using HCSA. First, 0.5 g of HCSA were dissolved in 20 mL of chloroform and then 0.4 g of PANI (EB) were added; the mixture was stirred for 18 h at 25 °C. Finally, HCSA-doped polyaniline (PANI-HCSA) was dried at room temperature.

PANI-HCSA (in the following indicated only as PANI) and copolymers, whose proportions are indicated in Table 1, were dissolved in chloroform under stirring for 4 h at 25 °C. Doped samples with graphene and iron oxide were obtained by adding the precursor oxides in the PANI/chloroform solution and stirred for 1 h at 25 °C. Finally, the copolymer was added and the solution stirred in the same conditions of the previously realized samples. Solutions viscosities were measured using a DVII rheometer, using a shear rate of 1 rad/s.

A 10 mL syringe coupled with a needle of 1.0 mm of diameter and a syringe pump set at a flow rate of 1.2 mL/h were used to electrospin polymer solutions. All the blended systems were homogeneous and no phase separation occurred before or during the electrospinning process. The electric field was generated using a high voltage supply. Fibers were synthesized using an applied voltage of 12.5 kV and a needle-to-collector distance of 15 cm and collected on aluminum foils. The electrospinning process was performed at 25 °C and with a relative humidity of 30%. A summary of the realized samples is reported in Table 1.

### 2.2. Characterization

Scanning electron microscopy (SEM) images were registered using a Phenom X-Pro microscope (Deben, Suffolk, UK) [32]. Samples were pasted on a copper foil and analyzed under an accelerating voltage of 5, 10 and 15 kV.

Thermogravimetric analysis and differential scanning calorimetry (TGA-DSC) were used to probe the thermal stability of the electrospun nanofibers. All measurements were performed using 10 mg of the sample under an inert atmosphere, from room temperature to 1000 °C with a heating rate of 10 °C/min. All experiments were performed twice, yielding similar results.

DC conductivity (σ) measurements were performed on freshly prepared samples (at least five samples), using a homemade device consisting of a counter electrode (CE) of platinum and a silver/silver chloride (Ag/AgCl) electrode as the reference electrode. Before and after carrying out conductivity measurements all samples were observed by electron microscopy to confirm that the measurement procedure did not affect fibers’ morphologies. The electrochemical impedance of samples was measured using the experimental set-up illustrated in Figure S1, with a three-electrode configuration, using an AMEL7050 (Milano, Italy) potentiostat-galvanostat coupled with AMEL7200 (Milano, Italy) frequency response analyzer. Work electrode (WE) was made using a bakelite base on which polymer fibers were fixed. Platinum wire was used as the counter electrode (CE) and silver/silver chloride (Ag/AgCl) electrode was used as reference. A 0.1 M ammonium acetate aqueous solution was used as an electrolyte. Electrochemical impedance spectroscopy (EIS) measurements were registered with a frequency range between 700 kHz and 100 Hz with an AC signal of 5 mV amplitude.

## 3. Results and Discussion

As to improve the production of self-standing thin films of highly conductive fibers, different aspects were considered. In particular, the copolymer type, the influence of polymers concentration and their molecular weight on the electrospun samples, together with mats thermal stability were all analyzed. Electrical properties, such as conductivity, specific capacitance and electrochemical performances were also evaluated and compared for all the realized samples.

Firstly, an attempt to produce pure PANI fibers was made but, without the addition of an auxiliary polymer to PANI in the precursor solution, no fibers formation occurred, as already evidenced in our previous work [33] and by other authors [34]. The electrospinning of neat PANI-solution resulted in the spraying of agglomerated droplets, as visible in the SEM image (Figure S2). A very large distribution of agglomerates from micro- to nanoscale was detected. Similarly, no fibers formation occurred even upon varying process parameters, such as the applied voltage, the flow rate solution and the needle-to-collector distance. This was due to the low processability of neat PANI polymer solution caused by the inherently poor solubility of PANI in common solvents [35] and the intrinsically low molecular weight of PANI, which did not allow the solution to reach a sufficient elasticity to be directly electrospun into a fiber form [36,37]. Therefore, two different copolymers, PVAc and PMMA were considered to realize polymer mixtures able to improve the processability of PANI for the electrospinning process.

**Figure 1:**
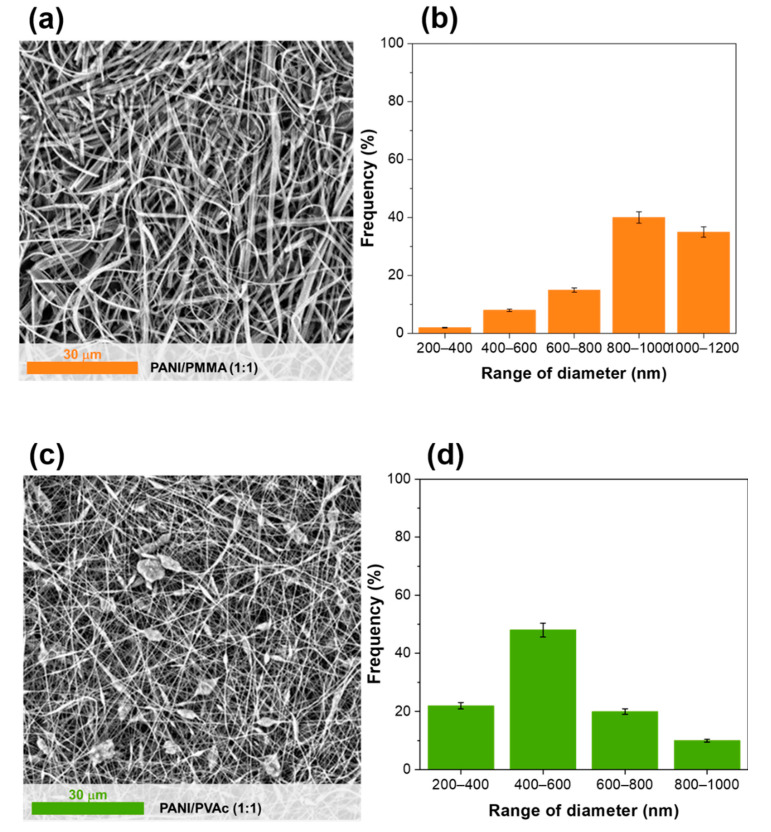
SEM images of (**a**,**b**) PANI/PMMA(1:1) nanofibers and distribution of dimensions and (**c**,**d**) PANI/PVAc (1:1) nanofibers and distribution of dimensions.

In both cases, by the visual inspection, it was possible to asses that using PMMA (sample PANI/PMMA (1:1), Figure S3a) and PVAc (sample PANI/PVAc (1:1), Figure S3b), self-standing mats were obtained with green coloring, undoubtedly attributable to the emeraldine salt form of polyaniline. At the microscopic level, in all mats produced the absence of preferential orientation was detected. Indeed, electrospun fibers appeared randomly arranged and their morphologies and diameters resulted highly affected by the type of utilized copolymer, as shown in Figure 1.

The use of PMMA (sample PANI/PMMA (1:1), Figure 1a) allowed one to obtain ribbon-like fibers with a narrow distribution of transversal dimensions. The ribbon-like fibers thickness was typically about 50 nm and rather uniform, whereas the width varied from the nanometer to the microscopic scale (Figure 1b). The formation of ribbon-like structures was caused by the rapid evaporation of the solvent and the consequent formation of a thin solidified surface layer (“skin”) in the propagating polymer jet. The latter, in turn, due to the atmospheric pressure, collapsed once formed and as a result, the circular cross-section became at first elliptical and then flat, causing the ribbon-like morphology [38,39].

Instead, with the addition of PVAc (sample PANI/PVAc (1:1), Figure 1c) a beady shape morphology along with thick fibers was obtained, showing an average diameter below  500 nm. The beady structures started to form in the middle of the fibers because of the surface tension that acted minimizing the total surface free energy [40]. Although beads formation is generally not advisable, a positive effect could be advantageous depending on the specific application, as previously observed by other authors [40].

In agreement with previous observations [41], the polymer concentration in the solution affected the morphologies of mats. In particular, if it is too low, fibers are not formed since the jet breaks up into droplets (electrospray). On the contrary, if it is too high, the electrospinning is suppressed because the continuous flow of the polymer solution to the capillary tip is prohibited. The effect of PANI concentration was evaluated with both the copolymers. It was observed that an increase of PANI in the polymer solution with PMMA promoted the formation of minor elongated structures (Figure S4b, PANI/PMMA (3:1)); whereas, in the polymer solution with PVAc, the obtained mats exhibited greater beads and irregular agglomerated formations (Figure S5b, PANI/PVAc (2:1)). The molecular weight of the copolymer used for the electrospinning also affected fibers morphologies [41]; generally speaking, the high molecular weight promoted fiber formation, whereas, when the molecular weight decreased, fibers decreased their size until they collapsed into particles (Figure S5a, PANI/PVAc_LMW_ (1:1)). Overall, as expected, the viscosity of the prepared polymer solutions increased according to both the increase of polymers molecular weights and solution concentration, as shown in Table 1.

**Figure 2:**
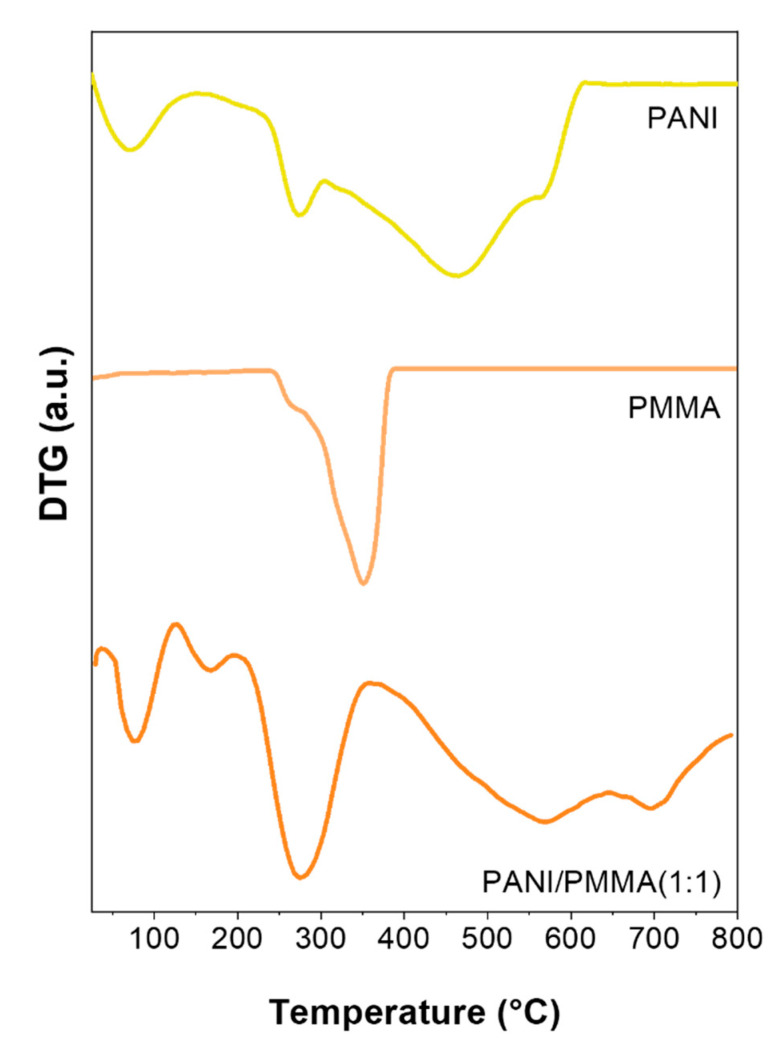
DTG profiles of different electrospun mats: PANI, PMMA and PANI/PMMA (1:1).

**Figure 3:**
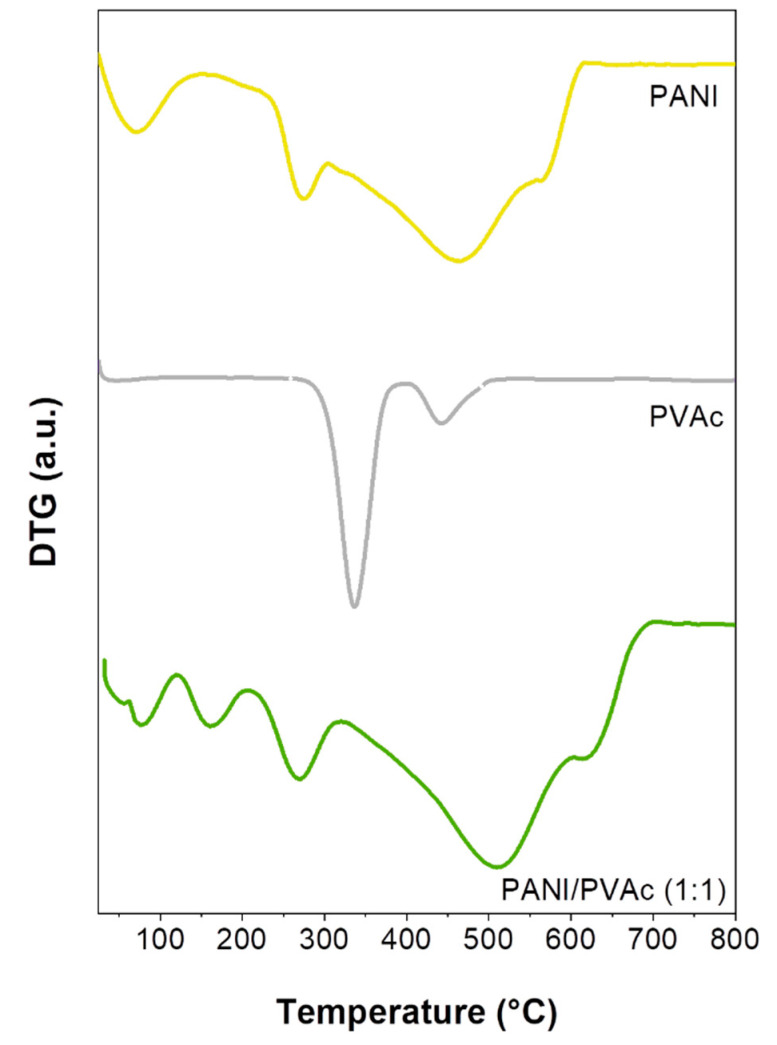
DTG profiles of different electrospun mats: PANI, PVAc and PANI/PVAc (1:1).

The thermal stability of blended mats was further investigated to explore their potential applications particularly directed to the development of electronic devices, where temperature-dependent characteristics maybe required. Figure 2 and Figure 3 show the DTG- curves for pristine electrospun polymers and blended systems; the derivative profiles provide a better understanding of the thermal decomposition behavior. For pure electrosprayed PANI (Figure 2 duplicated in Figure 3) three weight losses were recorded. The first one centered around 75 °C was attributable to the removal of residual humidity and solvent, the second peak was related to the dissociation of sulphonic groups from the polymer backbone and finally, the third one, beginning at around 350 °C, was related to a dramatic weight loss caused by the structural decomposition of the backbone. Both blended systems exhibited similar thermal behavior; upon the comparison with pure PANI, PANI/PVAc (1:1) and PANI/PMMA (1:1) showed superior stability. In fact, the higher weight loss was shifted to higher temperature values (peaks centered at 569 °C for PANI/PMMA (1:1) and 506 °C for PANI/PVAc (1:1)) revealing a good thermal resistance in the case of electronic devices use. The new peak in the blended profiles centered at around 170 ± 5 °C was likely due to the decomposition of the interfacial bonding between functional groups of PANI and the copolymer. Notwithstanding, the initial thermal decomposition, higher than 150 °C allowed to obtain a sensitive layer, characterized by good repeatability and long-term stability, suitable to be potentially used in gas sensing devices.

Finally, the effect of the presence of the copolymer in the blended systems together with the PANI content was also investigated in terms of conductivity, specific capacitance and electrochemical performances.

**Figure 4:**
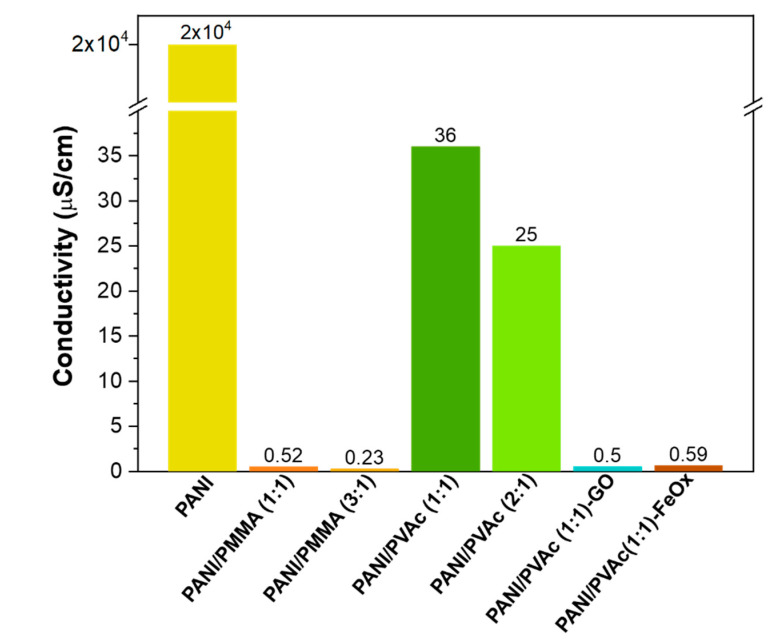
Electrical conductivity of as-electrospun mats.

Conductivity values of PANI blended mats were registered and compared with the pure electrosprayed PANI. Obtained values are reported in Figure 4. The conductivity of pure PANI mats was 2 × 10^−2^ S/cm, a value significantly lower than the best value (600 S/cm) for the pure PANI film reported by Zhang et al. [24]. However, it has been extensively demonstrated that the conductivity of PANI pure and/or blended is affected by numerous parameters, such as crystallinity degree, molecular weight, humidity level, branching presence, type of measurements, nature and amount of dopants [42,43].

PANI mats blended with both PMMA and PVAc apparently exhibited conductivity values several orders of magnitude lower than pure PANI mats. Anyway, these values were included in a broad interval, ranging from 10^−10^ to 0.1 S/cm, and referred to the values of recent works about the blended mats of PANI [44]. As expected, the electrical conductivity value registered for the electrospun polyaniline-blended fibers increased accordingly to the weight percent of doped polyaniline in the fibers. Electrical conductivities of PANI-PVAc (1:1) were two orders of magnitude higher than those of PANI/PMMA (1:1), being equal to the weight percent of PANI, indicating that copolymers are not simply acting as non-conductive fillers in fibers. The difference in conductivities of PVAc and PMMA blends can be attributed to the different intrinsic conductivities of PVAc (10^−6^ S/cm) and PMMA (10^−10^ S/cm) [30] and to the difference in their degree of compatibility with PANI [45]. Specifically, variations in conductivity values, with the same weight percentage of PANI in blends, could be mainly ascribed to the diverse arrangement of the copolymer (PVAc or PMMA) and PANI in nanofibers, and to the variation in dimensions and morphologies of electrospun mats [45].

The specific capacitance of both blended systems PANI/PMMA and PANI/PVAc was calculated from the CV result using Equation (1):

(1) $$C=\frac{AV_{2}-AV_{1}}{2mk\left(V_{1}-V_{2}\right)}\;\left[\frac{F}{g}\right]$$

**Figure 5:**
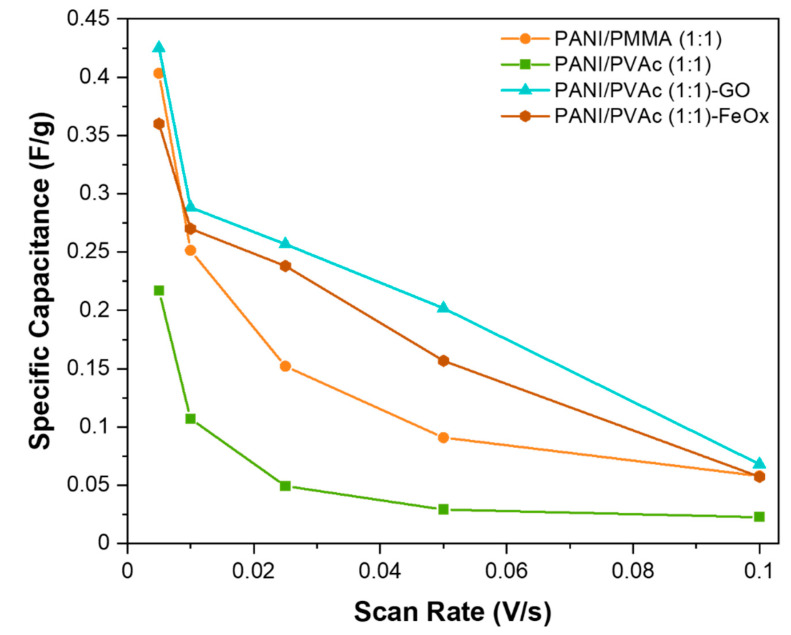
Specific capacitances of electrospun blended mats.

In the Equation “*AV*” is the area between the curve of charge-discharge, ‘*k*’ is the scan rate (V/s), ‘*V*’ is the potential window (V) and ‘*m*’ is the mass of the electrode material (g). The specific capacitances varying the scan rate are shown in Figure 5 for PANI/PVAc (1:1) and PANI/PMMA (1:1) systems respectively.

When the voltage scan rate was increased, a decrease in specific capacitance was recorded in all samples to a different extent. The maximum capacitance retention of 89% was observed in the sample PANI/PVAc (1:1), upon varying the current from the minimum to the maximum value; the corresponding value for PANI/PMMA (1:1) was 85%. This limited gap can be explained by two factors that balance each other: on one hand the higher value of conductivity of PANI/PVAc (1:1) with respect to PANI/PMMA (1:1), which decreases the ohmic loss, and on the other hand the elongated morphology of PANI/PMMA (1:1) fibers, which means shorter diffusion pathways inside fibers. The change in capacitance was similar to that observed in the case of PANI films, due to the expansion of the lattice and the excitation of charged carriers present at the imperfection sites or due to dipoles orientation [46].

**Figure 6:**
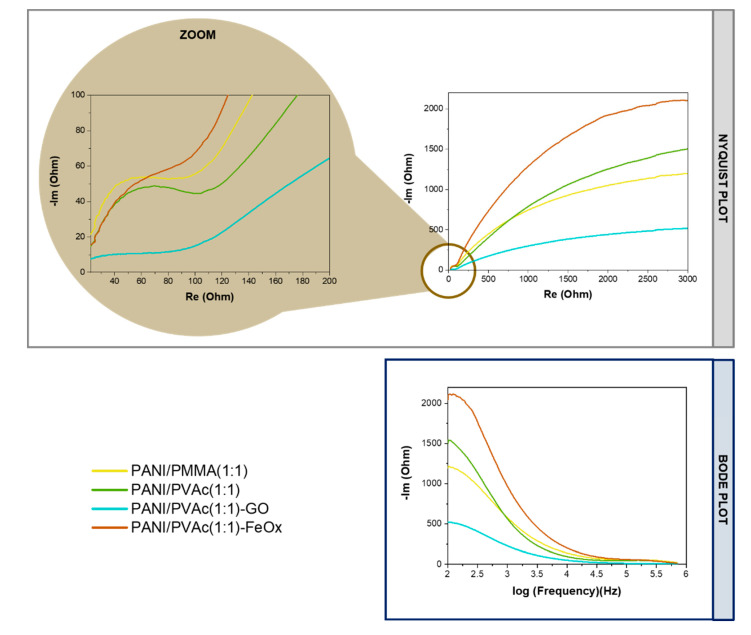
Impedance spectra of electrospun blended mats: Nyquist and Bode plot.

The electrochemical performances of PANI blended systems were further investigated by the electrochemical impedance spectroscopy (EIS) technique. Bode and Nyquist plots of bent samples show the high-, mid- and low-frequency regions, corresponding to the electrotransfer limited process, diffusion-limited electrode process and capacitive behavior (Figure 6), respectively. In the high-frequency region all impedance curves presented a semicircle. This impedance arc was due to the interaction at the interface between polymeric mats and electrolyte [47] at the low frequencies. For all samples, a deviation from the ideal vertical line caused by the irregularity of the polymeric mats was detected [48].

Equivalent circuit (Figure S6) was used to model the EIS experimental data: R_e_ is referred to the bulk electrolyte resistance, C_c_ and R_c_ are the polymeric mats layer capacitance and resistance respectively, Cdl is the double-layer capacitance, R_ct_ is the charge-transfer and σ is the coefficient of Warburg [49]. Respective values are reported in Table 2. The R_e_ value for PANI/PMMA (1:1) was higher than that of PANI/PVAc (1:1), proving that a higher R_e_ value was associated with greater compactness of mats [49]. In our samples, scanning electron micrographs supported this interpretation. C_c_ could be correlated to the absorption ability of mats [49]. The lower value of PMMA could be attributed to a higher hydrophobicity behavior of PMMA compared to PVAc. No significant differences in the R_c_ value were detected for the systems PANI/PMMA(1:1) and PANI/PVAc (1:1), being this value representative of the resistance of mats to the electrolyte penetration. In agreement with the literature, C_d_ value was at least one order of magnitude higher than C_c_ [50], for both systems. However, the value for PANI/PVAc (1:1) was twice compared to PANI/PMMA (1:1). Likely, this could be ascribed to the higher electronic conductivity of PANI/PVAc that in turn enabled a substantially higher fraction of the polymer mats to become electrochemically active, i.e., to form an active double layer in contact with the solution [51].

C_dl_ was in parallel with a kinetically controlled charge transfer resistance (R_ct_). As shown in Table 2, a marked increase in the R_ct_ of PANI/PVAc (1:1) was observed with respect to PANI/PMMA (1:1). The result indicated that the incorporation of PVAc into the PANI matrix caused a higher charge transfer resistance than those in the PANI blended with PMMA. A proposed explanation of this finding could be attributed to the interaction between the two polymers, PANI and PVAc. In other words, the methyl group in PVAc was active and could have many valence electrons (normally 6, 7, or 8) that, interacting with the group of functionalized PANI, led to a semiconductive behavior [52].

The coefficient of Warburg was similar for both blended systems, highlighting the same driving force of ionic transport in the blended mats [53]. However, further efforts should be devoted to a more in-depth maximization of interpretation of experimentally obtained data, trying to minimize expectations and predictions based on equivalent circuits [54].

According to the morphological and electrical–chemical properties discussed earlier, PANI/PVAc fibers demonstrated overall enhanced performances than the PMMA system.

**Figure 7:**
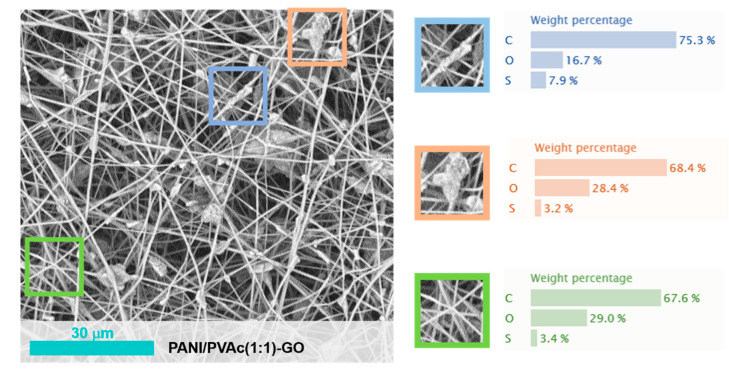
SEM images of PANI/PVAc (1:1)-GO and delimited EDX profiles.

**Figure 8:**
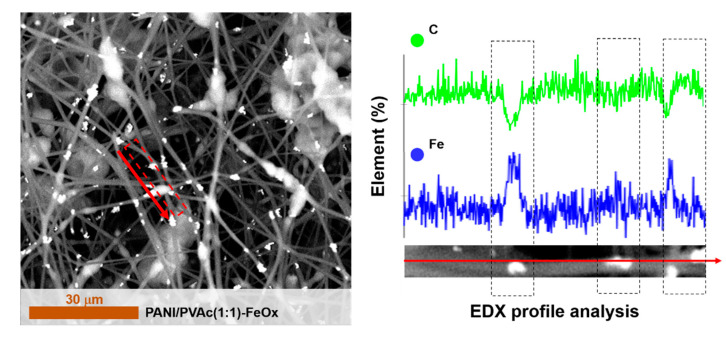
SEM images of PANI/PVAc (1:1)-FeO_x_ and delimited EDX profiles.

Currently, we are addressing our efforts to improve the electrochemical properties of doped PANI/PVAc fibers by adding graphene oxide and iron oxide. Indeed, until currently, PANI has already been combined with carbon materials to reinforce its stability and maximize its capacitance value [55], being known carbon behavior [56]. Moreover, oxide nanoparticles, as iron oxide, are widely used with conductive polymers for the development of energy storage materials, due to their attractive electrochemical properties, high specific theoretical capacitance, high efficiency of electrochemical energy conversion and controllable preparation [57]. The addition to PANI of graphene oxide, PANI/PVAc (1:1)-GO and iron oxide, PANI/PVAc (1:1)-FeO_x_, does not modify the rounded morphology of fibers (Figure 7 and Figure 8). We observed a minor presence of beady structures due to the higher viscosity of the precursor solution added with oxide particles (see Table 1). In both samples, the presence of agglomerates on the surface of the fibers was detected. Indeed, in the iron oxide case, agglomerates were clearly identified with the inorganic particles, due to EDX analysis (Figure 8).

The addition of oxides (both graphene and iron) promoted a loss in the conductivity (Figure 4), contrary to previous experimental observations [58,59]. This phenomenon could be attributed to the preparation methods of blended systems [60]. Indeed, as confirmed by microscopic analysis (Figure 7 and Figure 8), the addition of oxide particles favored the formation of islands of agglomerates apparently arranged on the surface of the polymeric fibers. Therefore, the overlapping of agglomerates on fibers resulted in a detrimental effect on the conduction pathways. However, further studies are necessary in order to fully address interfacial mechanisms.

Moreover, oxides addition led to a decrease in the specific capacitance while increasing voltage scan rate (Figure 5). PANI/PVAc (1:1)-GO and PANI/PVAc (1:1)-FeO_x_ exhibited higher values, although of the same order of magnitude, compared to the sample without additives. However, the specific capacitance registered for PANI/PVAc (1:1)-GO was lower compared to PANI/carbon-based composites obtained by electrochemical polymerization [61] or by in situ polymerization of monomer in the presence of graphene oxide [58]. Similarly, PANI/PVAc (1:1)-FeO_x_ specific capacitance was lower than PANI/iron oxide composites obtained by electrochemical synthesis [62]. These results could be explained considering both the diluent effect of the copolymer on pristine PANI and the electrospinning conditions adopted for the addition of oxide particles that caused, in both cases, the synthesis of intercalated mixtures of different materials rather than a nanocomposite material of blended PANI/PVAc doped with oxide particles.

Regarding the EIS, experimental data were fitted with the same circuit proposed for non-added mats (see Figure S6 and Table 2). The addition of graphene oxide to PANI/PVAc did not change significantly the R_e_, C_c_, R_c_ and C_dl_ values. The observed increment of R_ct_ was probably due to the presence of graphene oxide, causing a more inhomogeneous and disordered movement of charged carriers through polymer chains. Consequently, PANI/PVAc (1:1)-GO had a poor charge transport mechanism. Instead, in agreement with Arjomandi et al. [63], the addition of iron oxide enhanced a decrease in C_dl_ and R_ct_ values, compared to the non additive sample. Moreover, the linear part in the Nyquist plot (Figure 6), representing the Warburg coefficient, was much steeper for PANI/PVAc (1:1)-FeO_x_ compared to those of the other two electrode materials, reflecting its lower diffusion resistance and higher ion accessibility [64].

## 4. Conclusions

The effects of solution properties on the production of PANI-blended nanofibers by electrospinning were investigated. In particular, PMMA and PVAc were employed to improve PANI processability. An in-depth study was performed in order to understand their influence on both the structural and surface properties of the realized conductive thin films. Samples main electrical characteristics, as conductivity, specific capacitance and electrochemical performances were tested and discussed. The obtained mats were potentially addressed to numerous practical applications, including smart textiles, active sensors layers and electronic devices, such as supercapacitors.

This preliminary investigation reveals the following results:

- PVAc and PMMA were used to improve the electrospinning of PANI. Copolymers endowed samples with different morphologies but similar thermal stability;
- High content of doped PANI were used, thus preserving its main electrical characteristic even in a blended form;
- Mats obtained with PMMA showed an electrical conductivity value two orders of magnitude lower than the sample obtained with PVAc;
- Better capacitive performance of the PANI/PVAc sample was also observed with respect to the PMMA system;
- By increasing the copolymer quantity, a significant decrease in conductivity was observed. A similar trend was detected also for the specific electrical capacity, even if in a less amplified way;
- The addition of graphene and iron oxides was found to decrease mats conductivity.
- Finally, PANI/PVAc system appears to be the most promising sample. Notwithstanding these preliminary results, many efforts should be further devoted to adjust the experimental parameters of the electrospinning procedure in order to obtain a PANI/PVAc nanocomposite, optimally doped with oxide particles and characterized by improved morphology, electrical properties and performances.

## Figures and Tables

**Table 1:** List of samples electrospun, viscosities of solution, morphologies of yarns.

Code	Copolymer		Ratio PANI/Copolymer	Ratio Dopant/Solution	Viscosity (cPoise)	Morphology of Mats	Conductivity of Mats (μS/cm)
	Type	Molecular Weight					
PANI	-	-	-	-	-	Particles electrosprayed	2 × 10^4^
PANI/PMMA (1:1)	PMMA	960,000	1:1	-	1569 ±	Ribbons	0.52
PANI/PMMA (3:1)	PMMA	960,000	3:1	-	1187 ±	Cornflakes	0.23
PANI/PVAc_LMW_ (1:1)	PVAc	100,000	1:1	-	987 ±	No fiber formation	-
PANI/PVAc (1:1)	PVAc	500,000	1:1	-	1350 ±	Fiber beady	36
PANI/PVAc (2:1)	PVAc	500,000	2:1	-	1600 ±	Fiber beady	25
PANI/PVAc (1:1)-GO	PVAc	500,000	1:1	GO 2%	1450 ±	Fiber beady	0.50
PANI/PVAc (1:1)-FeO_x_	PVAc	500,000	1:1	Fe_2_O_3_ 2%	1460 ±	Fiber beady	0.59

**Table 2:** Values of the elements in equivalent circuits of Figure S6 fitted in the Nyquist diagrams of Figure 6.

Code	R_e_ (Ohm)	C_c_ (nF)	R_c_ (Ohm)	C_dl_ (nF)	R_ct_ (Ohm)	σ (Ohm⋅s^−1/2^)
PANI/PMMA (1:1)	42.86	7.44	117.30	83.72	204.20	45,070.00
PANI/PVAc (1:1)	27.14	10.68	107.30	174.70	693.80	45,430.00
PANI/PVAc (1:1)-GO	27.26	10.74	108.00	180.00	827.70	42,069.00
PANI/PVAc (1:1)-FeO_x_	24.84	10.44	129.60	54.06	543.40	85,657.00

## Supplementary Materials

The following are available online at https://www.mdpi.com/article/10.3390/nano11051269/s1, Figure S1: Scheme of the experimental set-up; Figure S2: Digital images of electrospun mats PANI/PMMA (1:1) and PANI/PVAc (1:1); Figure S3: Electrosprayed polyaniline PANI; Figure S4: Effect of PANI concentration in the solution to electrospun SEM images of (a) PANI/PMMA (1:1) and (b) PANI/PMMA (3:1); Figure S5: Effect of molecular weight of PVAc copolymer SEM images of (a) PANI/PVAcLMW (1:1) and (b) PANI/PVAc (2:1); Figure S6: Equivalent circuit.
