# Supplementary material for: Self Standing Mats of Blended Polyaniline Produced by Electrospinning
Source: Nanomaterials (Basel). 2021 May 12;11(5):1269. doi: 10.3390/nano11051269 (PMC8151508; doi:10.3390/nano11051269)

Supplementary Materials

Self Standing Mats of Blended Polyaniline Produced by Electrospinning

Antonio Fotia [1], Angela Malara [2,3,*], Emilia Paone [2,3], Lucio Bonaccorsi [2,3], Patrizia Frontera [2,3], Giulia Serrano [3,4] and Andrea Caneschi [3,4]

1 Department of Information Engineering, Infrastructures and Sustainable Energy, Mediterranea University of Reggio Calabria, Via Graziella Loc Feo di Vito, 89134 Reggio Calabria, Italy; antonio.fotia@unirc.it
2 Department of Civil, Energy, Environment and Material Engineering, Mediterranea University of Reggio Calabria, Via Graziella Loc Feo di Vito, 89134 Reggio Calabria, Italy; emilia.paone@unirc.it (E.P.); lucio.bonaccorsi@unirc.it (L.B.); patrizia.frontera@unirc.it (P.F.)
3 Consorzio Interuniversitario per la Scienza e la Tecnologia dei Materiali (INSTM), 50121 Firenze, Italy; giulia.serrano@unifi.it (G.S.); andrea.caneschi@unifi.it (A.C.)
4 Department of Industrial Engineering—DIEF, University of Florence, Via di S. Marta 3, 50139 Firenze, Italy
* Correspondence: angela.malara@unirc.it (A.M.)

List of Supplementary Information

Figure S1: Scheme of the experimental set-up.

Figure S2: Digital Images of electrospun mats PANI/PMMA (1:1) and PANI/PVAc (1:1).

Figure S3: Electrosprayed polyaniline PANI.

Figure S4: Effect of PANI concentration in the solution to electrospun SEM images of (a) PANI/PMMA (1:1), (b) PANI/PMMA (3:1).

Figure S5: Effect of molecular weight of PVAc co-polymer SEM images of (a) PANI/PVAc$_{LMW}$ (1:1), (b) PANI/PVAc (2:1).

Figure S6: Equivalent circuit.

Figure S1

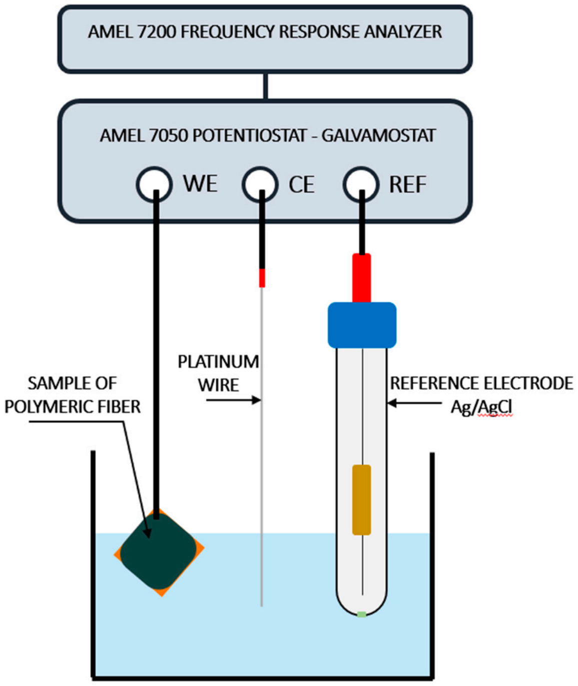
AMEL 7200 FREQUENCY RESPONSE ANALYZER
AMEL 7050 POTENTIOSTAT - GALVAMOSTAT
WE
CE
REF
PLATINUM WIRE
SAMPLE OF POLYMERIC FIBER
REFERENCE ELECTRODE Ag/AgCl

Figure S2

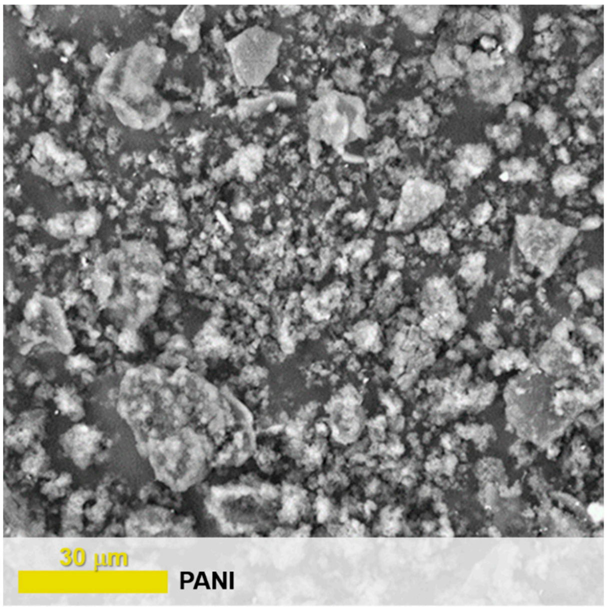

Figure S3

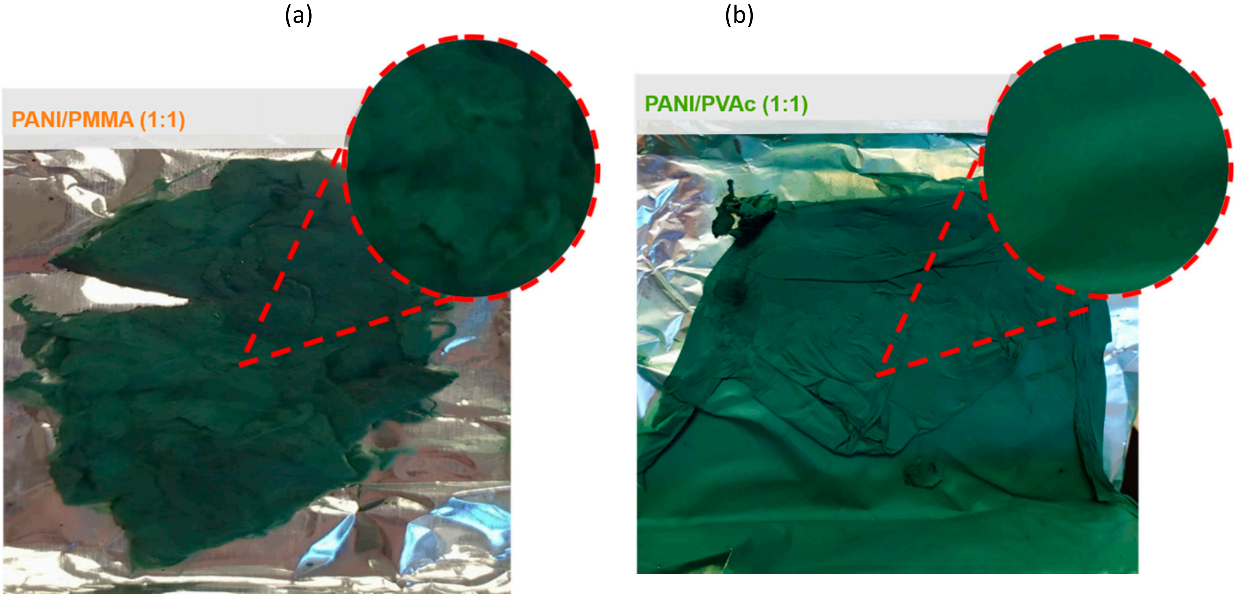

Figure S4

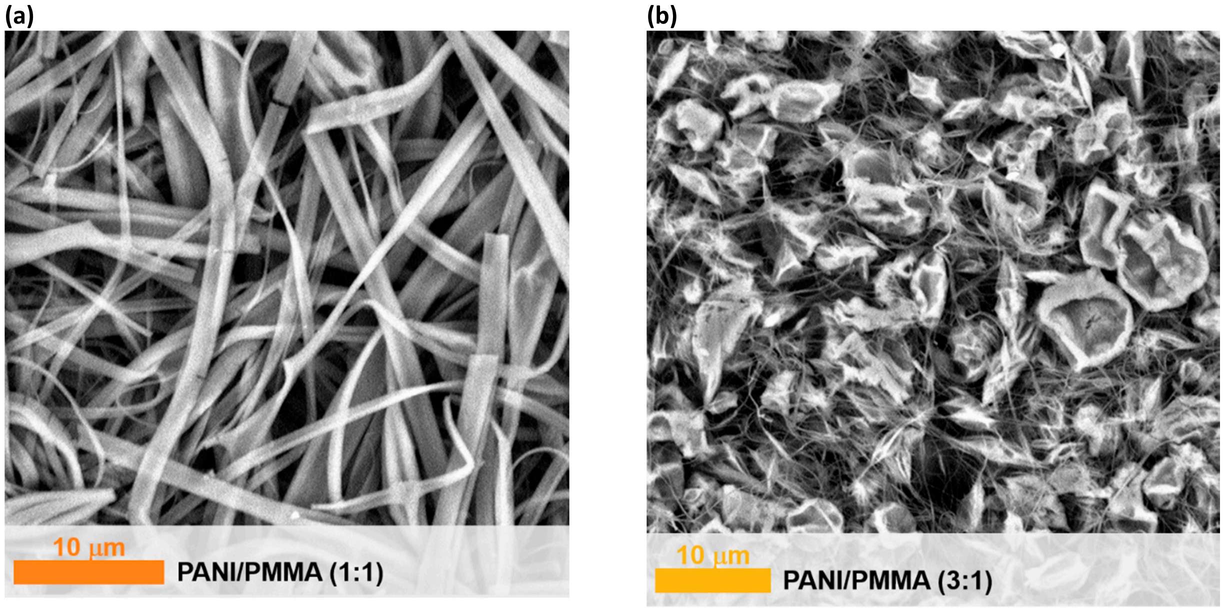

Figure S5

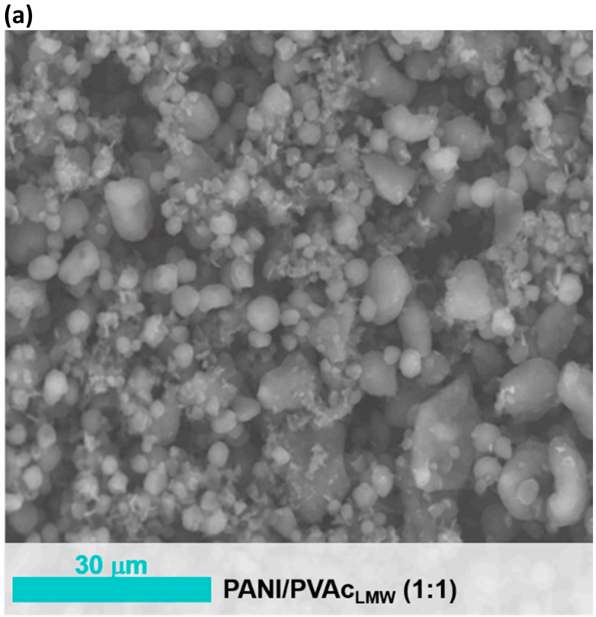

Figure S6

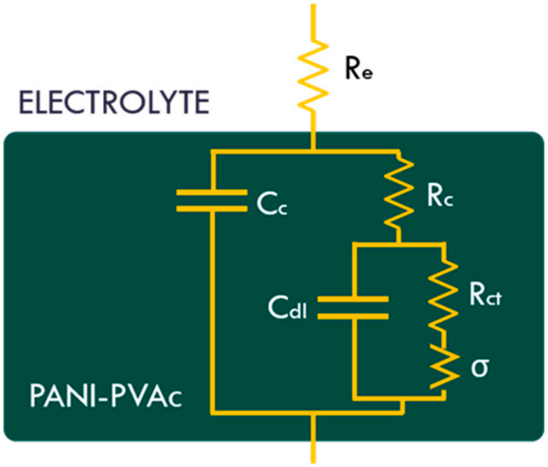

Supplement: Supplementary file 1 [file nanomaterials-11-01269-s001.zip › nanomaterials-1211444-supplementary.pdf]
